# A multi-centre open-label randomised non-inferiority trial comparing watchful waiting to antibiotic treatment for acute otitis media without perforation in low-risk urban Aboriginal and Torres Strait Islander children (the WATCH trial): study protocol for a randomised controlled trial

**DOI:** 10.1186/s13063-016-1247-y

**Published:** 2016-03-03

**Authors:** Penelope Abbott, Hasantha Gunasekera, Amanda Jane Leach, Deborah Askew, Robyn Walsh, Kelvin Kong, Federico Girosi, Chelsea Bond, Peter Morris, Sanja Lujic, Wendy Hu, Tim Usherwood, Sissy Tyson, Geoffrey Spurling, Markeeta Douglas, Kira Schubert, Shavaun Chapman, Nadeem Siddiqui, Reeion Murray, Keitha Rabbitt, Bobby Porykali, Cheryl Woodall, Tina Newman, Jennifer Reath

**Affiliations:** School of Medicine, Western Sydney University, Sydney, NSW Australia; University of Sydney, Sydney, NSW Australia; Menzies School of Health Research, Darwin, NT Australia; Discipline of General Practice, University of Queensland, Brisbane, QLD Australia; Southern Queensland Centre of Excellence in Aboriginal and Torres Strait Islander Primary Health Care (Inala Indigenous Health Service), Queensland Health, Brisbane, QLD Australia; University of Newcastle, Newcastle, NSW Australia; Indigenous Studies Research Network, Queensland University of Technology, Brisbane, QLD Australia; Centre for Big Data Research in Health, University of NSW, Sydney, NSW Australia; Sydney Medical School Westmead, University of Sydney, Sydney, NSW Australia; Awabakal Aboriginal Primary Health Care Centre, Newcastle, NSW Australia; Winnunga Nimmityjah Aboriginal Health Service, Canberra, ACT Australia; Aboriginal and Torres Strait Islander Community Health Services, Brisbane, QLD Australia; Tharawal Aboriginal Corporation, Sydney, NSW Australia

**Keywords:** Otitis media, Acute otitis media, Aboriginal and Torres Strait Islander peoples, Indigenous population, Children, Antimicrobial agents, Randomised controlled trial

## Abstract

**Background:**

Treatment guidelines recommend watchful waiting for children older than 2 years with acute otitis media (AOM) without perforation, unless they are at high risk of complications. The high prevalence of chronic suppurative otitis media (CSOM) in remote Aboriginal and Torres Strait Islander communities leads these children to be classified as high risk. Urban Aboriginal and Torres Strait Islander children are at lower risk of complications, but evidence to support the subsequent recommendation for watchful waiting in this population is lacking.

**Methods/Design:**

This non-inferiority multi-centre randomised controlled trial will determine whether watchful waiting is non-inferior to immediate antibiotics for urban Aboriginal and Torres Strait Islander children with AOM without perforation. Children aged 2 − 16 years with AOM who are considered at low risk for complications will be recruited from six participating urban primary health care services across Australia. We will obtain informed consent from each participant or their guardian. The primary outcome is clinical resolution on day 7 (no pain, no fever of at least 38 °C, no bulging eardrum and no complications of AOM such as perforation or mastoiditis) as assessed by general practitioners or nurse practitioners. Participants and outcome assessors will not be blinded to treatment. With a sample size of 198 children in each arm, we have 80 % power to detect a non-inferiority margin of up to 10 % at a significance level of 5 %, assuming clinical improvement of at least 80 % in both groups. Allowing for a 20 % dropout rate, we aim to recruit 495 children.

We will analyse both by intention-to-treat and per protocol. We will assess the cost- effectiveness of watchful waiting compared to immediate antibiotic prescription. We will also report on the implementation of the trial from the perspectives of parents/carers, health professionals and researchers.

**Discussion:**

The trial will provide evidence for the safety and effectiveness of watchful waiting for the management of AOM in Aboriginal and Torres Strait Islander children living in urban settings who are considered to be at low risk of complications.

**Trial registration:**

The trial is registered with Australia New Zealand Clinical Trials Registry (ACTRN12613001068752). Date of registration: 24 September 2013.

## Background

Acute otitis media (AOM) is a common reason for childhood presentation to health services and for prescription of antibiotics [1] and is commonly managed in general practice in Australia [2]. AOM creates a large health and cost burden for individuals and communities [1, 3]. Otitis media (OM) refers to inflammation of the middle ear space, which is characterised by the presence of middle ear effusion and causes illness and hearing loss [1]. In AOM there are also symptoms and signs of acute infection [4]. Serious complications of AOM, such as chronic suppurative otitis media (CSOM) (persistent ear discharge through a perforation in the eardrum) and mastoiditis, are rare in developed countries, where most cases of AOM resolve spontaneously [1, 5].

Antibiotics are not recommended for most children in developed countries with AOM due to limited clinical benefit [4] and the personal and public health risks of antibiotic resistance [6–8]. Aboriginal and Torres Strait Islander children who live in remote communities, where the incidence of AOM and prevalence of CSOM are high, are among those who are to expected benefit from antibiotic treatment for AOM [9–11]. However, there is little evidence for the appropriate management of AOM in the majority of Aboriginal and Torres Strait Islander children who live in urban settings and who are considered to be at lower risk of complications.

### Diagnosis and management of AOM

The particular combination of symptoms and signs of acute infection providing the most reliable diagnosis of AOM is debated. However, middle ear effusion accompanied by ear pain or bulging of the tympanic membrane is likely to be AOM [4].

High-level evidence from studies in developed countries shows that immediate antibiotic treatment confers only a modestly decreased duration of pain and fever at the cost of increased side effects [12–17]. International treatment guidelines [18, 19] currently recommend watchful waiting (initial observation and symptomatic treatment) and avoidance of immediate antibiotic treatment of AOM in children older than 2 years of age who are at low risk of complications. In this approach, carers are advised that children with persistent symptoms need clinical reassessment, at which point the clinician will review the need for antibiotics.

### OM and AOM management in urban Aboriginal and Torres Strait Islander children

Data on the burden of OM in Aboriginal and Torres Strait Islander children living in urban areas are sparse. Moderate to severe hearing loss was present in 32 % of 47 Aboriginal children and 7 % of 120 non-Aboriginal children aged 12 months or more in a study undertaken in an urban area of Western Australia [20]. In the most recent Australian Aboriginal and Torres Strait Islander Health Survey, 12 % of children under 14 years were reported to have ear or hearing problems, and this was the same in remote and non-remote areas [21]. Aboriginal and Torres Strait Islander children were significantly more likely than non-Indigenous children to be reported to have ear or hearing problems (rate ratio 1.3) [21]. As many Aboriginal and Torres Strait Islander Australians already experience marked disadvantage compared to other Australians, effective treatment of ear disease and prevention of hearing impairment in Aboriginal and Torres Strait Islander children is vital to maximise health and learning outcomes [22].

Recently, the recommended approach to the management of Aboriginal and Torres Strait Islander children with AOM has changed. The 2001 Australian guidelines [23] recommended immediate antibiotics for all Aboriginal and Torres Strait Islander children with AOM. However, the 2010 guidelines [5] recommend watchful waiting for Aboriginal and Torres Strait Islander children at low risk of CSOM. In these guidelines, Aboriginal and Torres Strait Islander children are classified as low risk if they are older than 2 years of age, do not have eardrum perforation or history of perforation and do not live in geographical areas with a high incidence of CSOM (defined as greater than 4 %), such as is consistently seen in remote Australia. As approximately 75 % of Aboriginal and Torres Strait Islander children live in urban and non-remote regional communities [24], where CSOM rates are likely to be less than 2 % [25, 26], this advice presents an important change in clinical practice [5].

### Pilot work undertaken prior to commencement of the study

An obstacle to evidence-based management of OM in primary care is lack of diagnostic accuracy [1, 27]. Diagnosis of AOM requires detection of middle ear effusion, which can only be reliably made using tympanometry or pneumatic otoscopy. However, both of these diagnostic aids are greatly underutilised by general practitioners (GPs), including in Aboriginal Medical Services [28]. Prior to the development of the WATCH study design, we undertook preliminary research concerning diagnosis of OM in the general practice setting [29, 30]. We determined that tympanometry was likely to be a more acceptable diagnostic technique than pneumatic otoscopy to aid GP diagnosis in AOM and incorporated this into the study design. We also undertook a retrospective medical record review in two participating services prior to commencement of the study to determine the numbers of children seen with AOM at these sites. This information was used to determine the study design and the duration required to meet recruitment targets.

### Study aims

The WATCH trial aims to determine whether watchful waiting is non-inferior to immediate antibiotic treatment in achieving clinical resolution of AOM without complications in Aboriginal and Torres Strait Islander children at low risk of complications who reside in urban areas. In addition to providing information to guide clinical management of AOM, we will investigate the relative costs and acceptability of the two treatment approaches to parents/carers and health care providers, and their views and experiences of the research processes.

## Methods/Design

### Primary objective

To determine whether watchful waiting is non-inferior to immediate antibiotic treatment in achieving clinical resolution of AOM without perforation at day 7 in urban Aboriginal and/or Torres Strait Islander children who are at low risk of CSOM.

### Secondary objectives

1. To determine whether watchful waiting is non-inferior to immediate antibiotic treatment in regards to symptom resolution, resolution of otoscopic signs, complication rates, and parent/carer satisfaction with treatment
2. To assess the cost-effectiveness of watchful waiting compared to immediate antibiotic prescription
3. To explore the acceptability of the two treatment arms and the experience of taking part in and conducting the trial, from the perspectives of parents/carers, health professionals and researchers, and to use this information to assist in the interpretation and translation of the findings

### Design

We are using a non-inferiority, open-label, randomised controlled trial (RCT) design. Non-inferiority studies seek to determine if a new treatment is no worse than a reference treatment by a predetermined margin. The new treatment is recommended if it is similar or better than the previous treatment, usually on the premise that the new treatment has other advantages over the reference treatment [31]. Children will be randomised to watchful waiting or immediate antibiotic therapy, stratified by study site and age of child (2 − 6 years and 7 − 16 years), using the National Health and Medical Research (NHMRC) Clinical Trial Centre Interactive Voice Response System (IVRS). It is not possible to blind the patient or treating staff. Outcome measurements will be verified by blinded assessment of tympanometry and video-pneumatic otoscopy (VO) data by an otolaryngologist and analysis will be blinded.

For practical reasons, the participating sites will commence staggered recruitment, commencing in two sites per block, 6 months apart. We will undertake cost-effectiveness analysis and a qualitative study in conjunction with the RCT as an integral part of the study design, with planning for both having commenced at inception [32]. A Data Safety Monitoring Board will oversee the conduct of the trial.

### Study setting

The study is a multi-centre study with six participating sites, all of which are in urban settings, including: five Aboriginal Community Controlled Health Services (ACCHSs); and one Indigenous Health Service. ACCHS are services dedicated to health care delivery for Aboriginal Australians and are run by the local Aboriginal communities for the local Aboriginal community and are also known as Aboriginal Medical Services. The Indigenous Health Service has different governance to the ACCHSs, being a state government-supported general practice, which provides primary health care to Aboriginal and Torres Strait Islander peoples. For the purposes of this publication the ACCHSs and the Indigenous Health Service are collectively referred to as health services. The health services are in three states in eastern Australia; two in Queensland (Brisbane), three in New South Wales (Newcastle and Sydney) and one in the Australian Capital Territory (Canberra).

Participants will be recruited from the clients attending the health services. We will obtain informed consent from each participant or their guardian.

### Aboriginal and Torres Strait Islander community ethical consultation and ethical approval

We are committed to conducting this research within the ethical framework recommended by the National Health and Medical Research Council’s Values and Ethics – Guidelines for Ethical Conduct in Aboriginal and Torres Strait Islander Health Research [33] and the Aboriginal Health and Medical Research Council’s key principles for ethical research [34]. We obtained approval from the Boards of the ACCHSs and the Inala Community Jury for Aboriginal and Torres Strait Islander Health Research (a group of Aboriginal and Torres Strait Islander people from the Inala community who guide all research conducted by the service) [35].

This study has been approved by the following ethics committees:

1. Aboriginal Health and Medical Research Council Ethics Committee (938/13)
2. Western Sydney University Human Research Ethics Committee (13/012032 | H10369)
3. Human Research Ethics Committee for the Northern Territory, Department of Health and Menzies School of Health (HOMER 13/2074)
4. Metro South Human Research Ethics Committee (Queensland Department of Health) (HREC/13/QPAH/366)
5. The University of Queensland Medical Research Ethics Committee (2013001093)

### Working with health services and communities

A strong tenet of this research is to work in a culturally safe and productive way with communities and health services, respecting their collaboration and providing them with adequate resourcing. We seek to build capacity both in terms of research skills and knowledge and skills in diagnosis and management of ear disease in these communities and health services. Services have nominated their own associate investigators (AIs) and we have provided funding for research officers (ROs) to be employed in each service. Aboriginal and Torres Strait Islander ROs help to ensure the research is locally culturally appropriate. Community research reference groups nominated by each health service have been funded and supported to provide ongoing community input to the research.

We are training all ROs and health service staff and providing support for professional development of the RO at each site. AIs and ROs are supported to attend annual investigator meetings. Training aims include promoting accurate diagnosis of AOM; the use of tympanometry and VO; evidence-based management of AOM; research skills and culturally appropriate research. The results of all aspects of the trial will be disseminated in each of the communities with which we are working.

### Intervention/Comparison

1. Watchful waiting: no immediate provision of antibiotic therapy at the time of enrolment. Subsequent treatment, including antibiotic prescription, is at the discretion of the treating physician
2. Antibiotic group: immediate prescription of antibiotic therapy (choice of antibiotic recommended to be based on Australian prescribing guidelines [36])

### Relevant concomitant care and interventions

Any concomitant care or intervention is permissible at the discretion of the treating physician and parent/carer. We will record analgesia and other symptom relief and concomitant care.

### Eligibility criteria

#### Inclusion

1. Aboriginal and/or Torres Strait Islander child (as documented by the health service)
2. Aged 2 years to 16 years (inclusive)
3. Not previously enrolled in the study
4. Current AOM without perforation diagnosed by the treating physician based on a Type B tympanogram and at least one of the following:
  bulging of the eardrum on otoscopy,
  ear pain (or irritability in 2 to 3 year-olds)

#### Exclusion

1. The child has been taking any antibiotic in the previous 4 days
2. At high risk of CSOM, as defined by residing in a geographic area known to have prevalence of CSOM greater than 4 %
3. A grommet in situ, or a current or past history of tympanic membrane perforation
4. A condition which increases the risk of complications (including immunosuppression, genetic or chromosomal abnormality, cleft palate or mid-face abnormalities such as seen in Down syndrome)
5. Systemic features necessitating antibiotic treatment (including septicaemia, meningitis, pneumonia, or urinary tract infection)

### Primary outcome

Proportion of children with clinical resolution of AOM, defined as all of the following: no pain, fever not higher than 38 °C, no bulging eardrum and no complications of OM (no perforation or mastoiditis) assessed by:

1. GP or nurse practitioner clinical examination on day 7 (acceptable range days 5 − 10)
2. Where 1. is not available, GP or nurse practitioner assessment of parental report and review of VO (recorded by trained RO), and no fever of at least 38 °C

### Secondary outcomes

1. Proportion of children with resolution of signs of AOM, through blinded otolaryngologist assessment of VO images and tympanometry taken at days 0 and 7* by a trained RO
2. Proportion of children with middle ear effusion, perforation and CSOM at week 7*, assessed by an independent blinded observer (otolaryngologist) reviewing VO and tympanometry data
3. Proportion of children with new antibiotic prescriptions (where ‘new’ is any antibiotic prescription provided after the recruitment consultation) for an index case of AOM assessed by review of medical record and by parent/carer report
4. Parent/carer-reported time to resolution of AOM symptoms assessed by parent/carer symptom report at days 3*, 7* and 14*, including the AOM Faces Scale [37]
5. Usage of analgesia for AOM symptom relief assessed by parent/carer report
6. Parent/carer satisfaction with AOM treatment assessed by parent/carer report using rating scale

(*acceptable ranges: days 2 − 4; days 5 − 10; days 11 − 17; weeks 6 − 8)

### Processes of enrolment and subsequent assessments

**Fig. 1:**
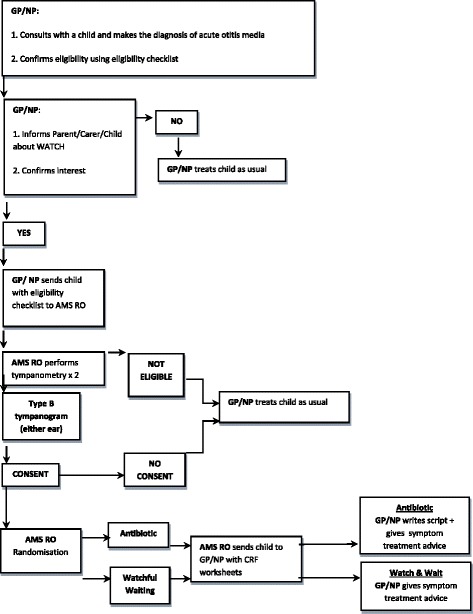
Participant enrolment flowchart

The enrolment processes are shown in Fig 1. Children will undergo subsequent assessments via phone or face-to-face at days 3, 7, and 14 and week 7. Medically-trained chief investigators will review participant medical records for any additional information relating to ear disease and/or its treatment which occurred in the 3 months following recruitment. Details of data collection at each of the scheduled assessment times are summarised in Table 1.

**Table 1:** Schedule of assessments – child/parent/carer

Assessment		Screening/Randomisation	Post-randomisation^a^				
	Performed by	Day 0	Day 3	Day 7	Day 14	Week 7	3 months
			(Days 2 − 4)	(Days 5 − 10)	(Days 11 − -17)	(Weeks 6 − 8)	(no window)
Clinical assessment	GP/NP	X		X			
Temperature	GP/NP/Health service RO or AI	X		X			
Tympanometry	GP/NP/AMS Health service RO or/AMS AI	X		X		X	
Digital video-otoscopy	GP/NP/Health service RO or AI	X		X		X	
Health history and socio-demographics	Health service RO/AI	X					
Symptoms questionnaire	AMS Health service RO/AMS AI	X	X	X	X		
Progress questions	AMS Health service RO/AMS AI		X	X	X	X	
Serious adverse events/severe study- related adverse events	AMS Health service RO/AMS AI		X	X	X	X	
Review Parent/Carer/Child Diary -symptom assessment using AOM-Faces Scale	AMS Health service RO/AMS AI		X	X	X		
Medical record review	Medical record reviewer						X

*AI* associate investigator, *AMS* Aboriginal Medical Service, *GP* general practitioner, *NP* nurse practitioner, *RO* research officer

^a^Bracketed day/week numbers in the Post-randomisation column above represent acceptable time ranges.

### Data collection and data management

Data collected during each study visit will be recorded using standardised case report forms (CRFs). Data from paper CRFs will be entered into an electronic database by the ROs at each of the participating sites, ideally within 2 weeks of data collection. Electronic CRFs will have participant identifying details removed and be labelled with coded identifiers including randomisation number. Initially, 10 % of paper CRFs will be crossed checked against electronic CRFs for accuracy by a Western Sydney University RO, with a more intensive review conducted as indicated. Data discrepancies will be discussed with and amended by the site accordingly. Randomisation allocation is generated and stored separately from the electronic database and will only be forwarded to the study statistician upon request.

### Sample size and power

With 198 children in each arm of the study, the study will have 80 % power to detect a non-inferiority margin of up to 10 % at a significance level of 5 %, assuming a clinical improvement rate of at least 80 % in both study groups [38]. Allowing for a dropout rate of up to 20 % at days 5 − 10, the total sample size required is 495 children.

### Analysis

The primary outcome measure is clinical resolution on day 7 as defined above. We will compare the proportion of children meeting the definition of clinical resolution using intention-to-treat analysis (ITT), as well as per-protocol analysis, as non-inferiority trials that use only ITT can be biased towards finding non-inferiority [39]. The per-protocol population will consist of all randomised patients who have adhered to the allocated treatment, were not lost to follow-up, and who have no major protocol deviations. All participants with complete outcome information at day 7 will be included. For protocol deviations where participant data are available, these will be analysed and the impact of their inclusion assessed and reported.

Sensitivity analyses will describe the impact of the following alternative assumptions: (1) missing = clinical failure; (2) missing = clinical success; (3) extrapolation from days 2 − 4 telephone interview; (4) best case for watchful waiting; and (5) worst case for watchful waiting. A secondary per-protocol analysis (accounting for non-adherence and change in treatment) will be done to describe any short-term benefits and harms associated with antibiotic use.

Non-inferiority of watchful waiting compared with antibiotic treatment will be accepted if the lower bound of the 95 % CI around the estimated difference in the primary endpoint rates does not exceed 10 %.

Data analysis will include analysis using Fisher’s exact and Chi-square tests for categorical outcomes, and parametric and non-parametric tests for continuous measures, as required. The difference in the primary endpoint between the two groups will be expressed as a risk difference. Where appropriate, odds ratios (ORs) will be calculated and will include both unadjusted (crude) and adjusted ORs. Adjusted ORs will be obtained using multivariable logistic regression, adjusting for baseline covariates. Time to resolution of AOM symptoms will be modelled using a multilevel Cox (proportional hazards) regression analysis and graphically displayed using Kaplan-Meier curves. Secondary analyses by stratification factors (recruitment site and age) will be carried out, numbers permitting.

### Cost-effectiveness data collection and analysis

As a secondary outcome measure, we will assess the cost-effectiveness of watchful waiting compared to immediate antibiotic prescription, as measured through the incremental cost-effectiveness ratio (ICER). The ICER is defined as:

$$ "\mathrm{ICER}"=\left({\mathrm{C}}_{\_2}-{\mathrm{C}}_{\_1}\right)/\left({\mathrm{Q}}_{\_2}-{\mathrm{Q}}_{\_1}\right) $$

where *C*_*i*_ and *Q*_*i*_ denote costs and Quality-adjusted Life Years (QALYs) [40] associated with the treatment received in trial arm *i*, and *i* is 1 for antibiotic treatment and 2 for watchful waiting.

#### Costs of treatment

Costs of treatment including medication costs, health service usage and non-medical costs will be computed for events related to AOM and its treatment. This information will be collected as follows:

1. Medication costs will be calculated using parent/carer-reported antibiotic/analgesia usage collected in the CRFs
2. Health service usage data will be collected mostly in the CRFs, but also through medical record review. The medical record data will be reviewed within two distinct time periods: 0 to 7 weeks post recruitment and 7 weeks to 3 months post recruitment. Medical record data relating to weeks 0 to 7 will be compared with CRF data to check and enhance reliability of health service usage information. The medical record data from week 7 to 3 months will allow collection of later information, if any, relating to health service utilisation, such as later follow-up appointments relating to the ear disease or its complications. We will estimate costs of health service utilisation and complications by applying publicly available price factors to utilisation figures. Complications requiring a hospital admission will be priced using the National Public Cost Weight Tables [41]. Complications requiring a visit to a medical practitioner will be priced according to item numbers within the Australian universal health benefit scheme, Medicare
3. Non-medical costs will be calculated from parent/carer data collected in the CRF. We will use a societal point of view and, therefore, include non-medical costs, both tangible and intangible, borne by the carers of the child. The main non-medical tangible cost is transportation to and from the GP or any other health care provider. The main non-medical non-tangible cost is time spent by parents/carers in activities related to the AOM or its treatment, which take them away from their usual role or activities. The parent/carer estimate of the time spent on such activities will be converted into a monetary figure using standard guidelines for wages and productivity [42].

#### Quality-adjusted Life Years (QALYs)

We will calculate QALYs using CRF data from parent/carers. The CRF data will be used to measure the time a child spends in different health states associated with both the natural course of the disease (mostly characterised by pain), and complications (such as rash or gastrointestinal problems). Each health state will be assigned a utility value (that measures the degree of preference society places on that health state) using QALY weights found in the medical literature, since a novel evaluation of preferences is not in the scope of this trial. QALYs will then be computed by combining the information about the time spent in the different health states with the QALY weights.

Missing cost-effectiveness data will be managed through the use of imputation, rather than deleting observations. Sensitivity analysis is particularly important, since QALY weights currently available are not specific to the Aboriginal and Torres Strait Islander population. Sensitivity analysis will be performed using both one-way and multi-way Monte Carlo simulations, and will allow us to estimate how much the uncertainty regarding QALY estimates contributes to the overall results. In both cases the key output is the set of conditions under which the ICER remains in an acceptable range, in order for watchful waiting to be considered cost-effective.

### Qualitative data collection and analysis

We will undertake a qualitative study using thematic analysis of individual and group interviews to examine parents’/carers’, health professionals’ and researchers’ experiences of the trial and views on the different treatment approaches to AOM. Semi-structured interviews will be undertaken with consenting health care providers (Aboriginal health workers, nurses, GPs, allied health providers) and parent/carers in selected sites, including parent/carers who declined to take part in the RCT. Interview participants will be selected using a purposive sampling strategy for maximal variation of age, gender, study experiences and views [43]. Interviews will also be undertaken with consenting research officers and the community reference groups in each site. The interviews will be taped, transcribed, de-identified and coded. Transcripts will be coded by two members of the research team with contrasting disciplinary perspectives, and any differences in interpretation resolved by discussion, and a thematic analysis undertaken [44].

## Discussion

The WATCH trial will answer the important clinical question: whether watchful waiting is (or is not) inferior to immediate antibiotic therapy for urban Aboriginal children with AOM who are at low risk of complications. We will assess the cost-effectiveness of the two approaches and qualitatively examine the acceptability of the alternate approaches to management of AOM to parents and carers, and health care providers. Furthermore, we will examine experiences of the trial process itself in order to inform future RCT studies in Aboriginal health settings.

This trial will make an important contribution to the evidence base for the safe management of AOM in urban Aboriginal children. Australian guidelines for the management of AOM have recently been updated [5] and Aboriginal and Torres Strait Islander children living in urban settings are no longer classified as high risk for complications of AOM, and thus health care providers are advised they should be treated with watchful waiting. This requires a change in clinical practice. The expert consensus guideline advice has been extrapolated from studies in low-risk, developed countries internationally. There have been no studies into the relative effectiveness of a watchful waiting approach for urban Aboriginal and Torres Strait Islander children, nor of attitudes of carers and health care providers to this approach.

Research undertaken with Aboriginal and Torres Strait Islander people must have net benefits for communities, incorporate community research control, be conducted in a manner sensitive to the cultural principles of Aboriginal society, be appropriately resourced and enhance the skills and knowledge of the Aboriginal and Torres Strait Islander people, communities and organisations that are participating in the project [34]. This study has been designed with awareness and commitment to these guiding principles and to the flexibility required to undertake a randomised controlled trial in Aboriginal and Torres Strait Islander health settings.

Ear health is a core priority for Aboriginal communities and the health care providers who work with those communities. Aboriginal and Torres Strait Islander people experience marked disadvantage compared to other Australians [45] and effective treatment of ear disease and prevention of hearing impairment in Aboriginal children is vital to maximise health and learning outcomes [22]. The ultimate goal of this research is to ensure appropriate treatment of acute infections of the middle ear so as to maintain the ear health of Aboriginal children and decrease their risk of developing chronic complications.

### Trial status

The trial is registered with the Australia New Zealand Clinical Trials Registry (ACTRN12613001068752). Contracts have been signed with six participating health services, which were selected based on service size and geographic location. Four ACCHSs declined to participate in the trial when approached for potential participation. Training and orientation to the study has been undertaken in all sites and recruitment has commenced in five sites. There was a protocol change made after one child was recruited to the study. This comprised:

1. Revision of the diagnostic criteria of AOM to comprise ‘ear pain *or irritability*’ in a child aged 2 to 3 years)
2. Amendment to the primary outcome to allow the GP to assess the outcome (resolution of AOM) through assessment of parental report and review of VO- and RO-collected temperature if the child had not come in to see the GP
3. Removal of a requirement for tympanometry and VO at the day-3 and day-14 data collection points, allowing data to be collected by telephone rather than face-to-face

Another protocol change to eligibility inclusion criteria was made after 36 children were recruited to the study, permitting the eligibility diagnosis of AOM without perforation and the day-7 primary outcome assessment by the treating nurse practitioner as well as the GP. This was deemed necessary as it was the normal practice in one of the later recruiting sites for diagnosis and treatment of AOM to be determined by credentialed nurse practitioners at the health service.

## Abbreviations

ACCHS: Aboriginal Community Controlled Health Services
AI: associate investigator
AMS: Aboriginal Medical Service
ANOVA: analysis of variance
AOM: acute otitis media
CRF: case report form
CSOM: chronic suppurative otitis media
GP: general practitioner
ICER: incremental cost-effectiveness ratio
NP: nurse practitioner
OM: otitis media
QALYs: Quality-adjusted life Years
RCT: randomised controlled trial
RO: research officer
VO: video-pneumatic otoscopy
WATCH: Watchful waiting for Aboriginal and Torres Strait Islander Children with acute otitis media: the WATCH trial
